# Altered regional activity and inter-regional functional connectivity in psychogenic non-epileptic seizures

**DOI:** 10.1038/srep11635

**Published:** 2015-06-25

**Authors:** Rong Li, Yibo Li, Dongmei An, Qiyong Gong, Dong Zhou, Huafu Chen

**Affiliations:** 1 Key Laboratory for Neuroinformation of Ministry of Education, School of Life Science and Technology, University of Electronic Science and Technology of China, Chengdu 610041, PR China; 2 Department of Neurology, West China Hospital of Sichuan University, Chengdu 610041, PR China; 3 Department of Radiology, West China Hospital of Sichuan University, Chengdu 610041, PR China

## Abstract

Although various imaging studies have focused on detecting the cerebral function underlying psychogenic non-epileptic seizures (PNES), the nature of PNES remains poorly understood. In this study, we combined the resting state fMRI with fractional amplitude of low-frequency fluctuations (fALFF) and functional connectivity based on the seed voxel linear correlation approach to examine the alterations of regional and inter-regional network cerebral functions in PNES. A total of 20 healthy controls and 18 patients were enrolled. The PNES patients showed significantly increased fALFF mainly in the dorsolateral prefrontal cortex (DLPFC), parietal cortices, and motor areas, as well as decreased fALFF in the triangular inferior frontal gyrus. Thus, our results add to literature suggesting abnormalities of neural synchrony in PNES. Moreover, PNES exhibited widespread inter-regional neural network deficits, including increased (DLPFC, sensorimotor, and limbic system) and decreased (ventrolateral prefrontal cortex) connectivity, indicating that changes in the regional cerebral function are related to remote inter-regional network deficits. Correlation analysis results revealed that the connectivity between supplementary motor area and anterior cingulate cortex correlated with the PNES frequency, further suggesting the skewed integration of synchronous activity could predispose to the occurrence of PNES. Our findings provided novel evidence to investigate the pathophysiological mechanisms of PNES.

Psychogenic non-epileptic seizures (PNES) are paroxysmal episodes of disruptive changes in consciousness, cognitive control, emotional or behavioral functions that mimic epileptic events but lack electrophysiological discharges^1^. Patients with PNES may be misdiagnosed as suffering from intractable epilepsy, which easily cause unnecessary anticonvulsant medications and other iatrogenic consequences of treatments^2^. Although numerous studies have linked PNES to a high prevalence of comorbid psychiatric conditions^3^ to investigate the underlying psychological aetiology, the nature of PNES remains poorly understood.

The results of a number of previous studies have suggested that PNES are associated with the abnormal functional integration of a distributed network of brain regions^4^,^5^. An early electroencephalography (EEG) study involving the whole-head surface topography of multivariate phase synchronization (MPS) found decreased prefrontal and parietal synchronization in PNES, which reflect the dysfunction of fronto-parietal networks^4^. van der Kruijs *et al*. employed task-related functional connectivity (FC) analysis to show for the first time that PNES patients exhibit stronger connectivity values between the areas involved in emotion, executive control, and sensorimotor networks (SMN)^5^. In addition, several EEG-based graph theoretical modeling studies^6^,^7^ have investigated large-scale inter-regional functional correlations in PNES patients, and showed that PNES exhibit altered network properties in the form of small-worldness and clustering coefficients. Together, these studies pointed to an aberrant neural organization of network function in PNES. Despite these advances, little is known whether patients with PNES show local regional abnormalities in resting brain function.

In recent years, the resting state functional magnetic resonance imaging (rfMRI), with better spatial resolution and no radiation exposure, has been used as an effective technique in exploring regional and spontaneous neural functions in clinical studies^8^,^9^,^10^,^11^. Early in 1995, Biswal *et al*.^12^ discovered that the spontaneous low-frequency (<0.08 Hz) oscillations (LFOs) in the resting state blood oxygen level dependent (BOLD) were coherent among motor cortices. Subsequently, several methods have been developed to detect the amplitude of LFOs using rfMRI. One is referred to as the amplitude of low frequency fluctuations (ALFF)^13^, in which the square root of the power spectrum is integrated in a low frequency range, for detecting the regional intensity of spontaneous fluctuations in the BOLD signal. An improved approach is the fractional ALFF (fALFF)^14^, which significantly improves the sensitivity and specificity in detecting regional spontaneous brain activity, as the ratio of power spectrum of low frequency range to that of the entire frequency range was computed. To date, the fALFF method has been extensively employed in healthy subjects^15^ and clinical populations^16^.

Previous studies have demonstrated that PNES exhibit altered functional connections in the prefrontal and parietal cortices and SMN^4^,^17^; hence, we hypothesized that the patients with PNES would show abnormal fALFF in these regions. Moreover, even if abnormal fALFF is detected in PNES, we question that whether the specific regional signal fluctuation amplitude abnormalities are associated with potential connectivity alterations of that local activity across brain regions. The studies of FC and networks generally rely on the correlations and relative independence of low frequency fluctuation signals of rfMRI. However, the reliability of fMRI signals may be associated with the level of noises, as well as the meaningful neuronal functions^18^. Therefore, fluctuation amplitudes may indeed affect the FC. Recent studies have observed overlaps between abnormalities in regional ALFF and FC in several brain regions in affective disorder^19^, stuttering^20^, post-traumatic stress disorder^21^ and schizophrenia^22^. These studies suggested a relationship between ALFF and FC; however, both the regional cerebral function and functional integration have not been investigated in patients with PNES.

To address such issues, we utilized two rfMRI metrics to respectively characterize the regional changes and inter-regional functional connection in PNES. The fALFF measures the regional signals change of spontaneous activity within a specific frequency range, while the FC reflects the level of integration of that local activity across brain regions, which can provide new insights to the pathophysiological mechanism underlying PNES. Patients with PNES exhibit a well-established dysfunction in relative level of emotion regulation, executive control, and motor function^23^,^24^ because of altered fronto-parietal and SMN connections. Hence, we predicted that patients would show abnormal fALFF and functional integration in the frontal and parietal cortices and motor regions. Moreover, we further examined the correlations between the fALFF and FC values and the frequency of PNES.

## Results

### Regional cerebral function

**Figure 1:**
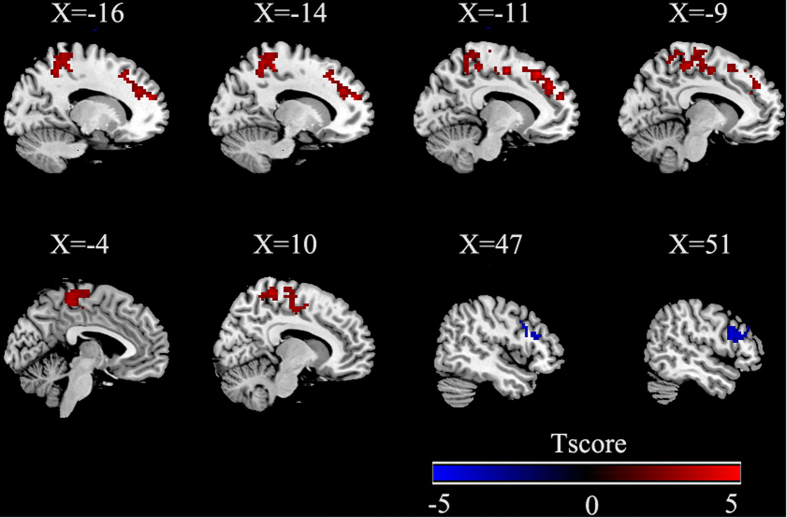
Group differences of fALFF using two-sample *t*-tests. Regions showing increased (red) and decreased (blue) fALFF values in patients with PNES compared to healthy controls. The results were corrected by AlphaSim(all the clusters survived *p* < 0.05, a combined threshold of p < 0.01 with a minimum cluster size of 75 voxels). Color bar indicates the T score. More details of altered fALFF regions are described in Table 2.

fALFF group differences are shown in Table 2 and Fig. 1 (p  < 0.05, AlphaSim corrected). Compared with the healthy controls (HC), PNES patients exhibited significantly increased fALFF in the left superior frontal gyrus (SFG), left precuneus, left paracentral lobule, right postcentral gyrus and left supplementary motor area (SMA). Patients showed decreased fALFF in a triangular part of the right inferior frontal gyrus (IFG).

### Inter-regional functional connectivity

**Figure 2:**
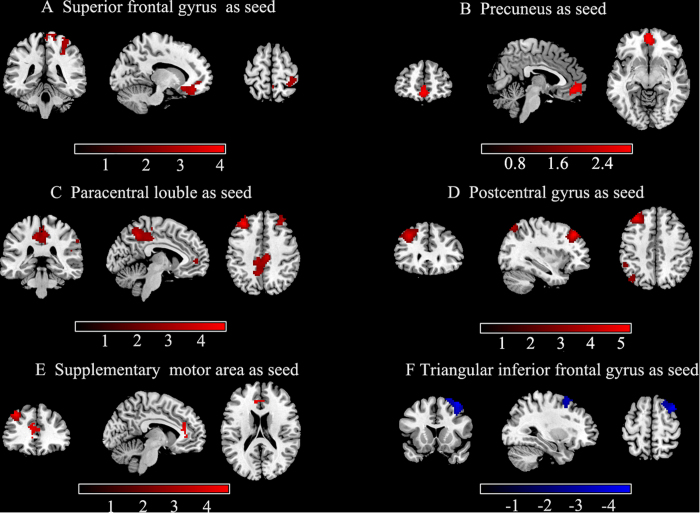
Group differences of inter-regional FC maps between seeds and the rest voxels of the brain. Six abnormal fALFF regions (superior frontal gyrus, precuneus, paracentral lobule, postcentral gyrus, supplementary motor area, and inferior frontal gyrus) were defined as seeds for FC analyses. Compared to healthy controls, patients with PNES showed increased (red) and decreased (blue) connectivity based on these seeds. The results were corrected by AlphaSim(all the clusters survived *p* < 0.05, a combined threshold of *p* < 0.01 with a minimum cluster sizes of 116,115,162,150,79 and 157 voxels, respectively). Color bar indicates the T score. More details of altered FC regions are described in Table 3.

Relative to HC, a significantly abnormal FC between 6 seeds and a number of cortical regions were observed. Table 3 and Fig. 2 show the results of the between-group FC comparison with the seeds (*p* < 0.05, AlphaSim corrected). For the seeds with higher fALFF value (left SFG, left precuneus, left paracentral lobule, right postcentral gyrus and left SMA), the PNES group displayed increased FC in the precuneus, anterior and median cingulate cortices, postcentral gyrus and particular regions of the frontal and parietal cortices. For the seed with lower fALFF value (right triangular IFG), we detected decreased FC in the middle frontal gyrus in patients with PNES.

### Correlation analyses

**Figure 3:**
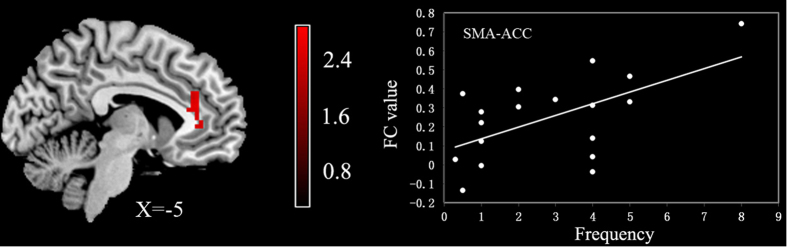
Correlation between the frequency of PNES attacks per month and FC values. The mean *z* values of left ACC based on the SMA seed were significantly positively correlated with the frequency of PNES (*r* = 0.58, *p* < 0.05). ACC, anterior cingulate cortex; SMA, supplementary motor area.

The frequency of PNES was not correlated with the fALFF values in any of the seed regions. However, we observed a significant correlation between the mean FC values and the frequency of PNES. As shown in Fig. 3, the FC between SMA and left anterior cingulate cortex (ACC) was positively correlated with the frequency of PNES (*r* = 0.577, *p* < 0.05). In addition, there was no significant association between the fALFF values and altered connectivity *z* scores in any areas with significant connectivity alterations.

### Reproducibility

**Figure 4:**
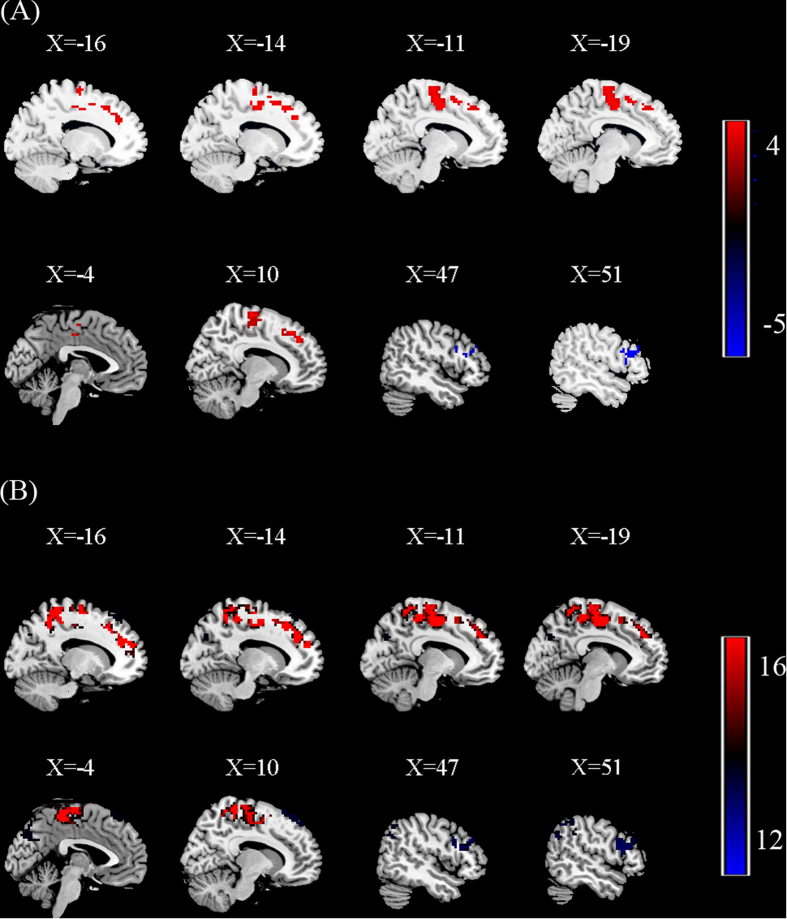
(**A**) Split-half sample reproducibility. Group differences of fALFF were performed between 9 PNES patients and 10 healthy controls (HC) using two-sample *t*-tests. The results were corrected by AlphaSim. Regions showing increased (red) and decreased (blue) fALFF values in PNES compared to HC. Color bar indicates the T score. (**B**) Leave-one-out sample reproducibility. The group comparisons based upon the permutated samples (i.e., 17 PNES vs. 20 healthy controls) for total 18 times. For each voxel, the color bar indicates number of tests where this voxel exhibited significant group differences across the total 18 tests.

**Figure 5:**
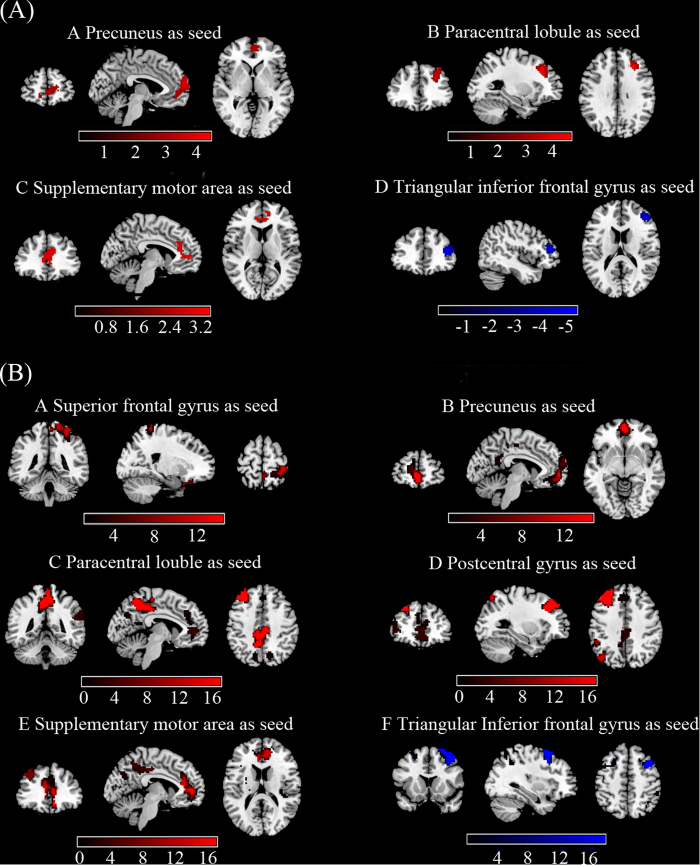
(**A**) Split-half sample reproducibility. Group differences of FC were performed between 9 PNES patients and 10 healthy controls (HC) using two-sample *t*-tests. The results were corrected by AlphaSim. FC patterns seeded only in left precuneus, left paracentral lobule, left supplementary motor area and right inferior frontal gyrus were highly reproducible, including increased (red) and decreased (blue) connectivity. Color bar indicates the T score. (**B**) Leave-one-out sample reproducibility. The group comparisons based upon the permutated samples (i.e., 17 PNES vs. 20 healthy controls) for total 18 times. For each voxel, the color bar indicates number of tests where this voxel exhibited significant group differences across the total 18 tests.

To test the reproducibility of our findings, we carried out both split-half and leave-one-out validations as suggested by a previous study^25^. For the split-half analysis, 9 PNES patients and 10 healthy controls were randomly selected from the full sample and underwent the same statistical comparison of fALFF. The results are illustrated in Fig. 4A, which replicated most of the findings from the full sample (*p* < 0.05, AlphaSim corrected). Subsequently, seed-based FC analysis was performed in the other half of the sample. The results showed that the overall previous FC findings (Fig. 2) were partly limited to the spatial extent of the half sample (Fig. 5A, *p* < 0.05, AlphaSim corrected). Visual inspection indicated that the FC patterns seeded only in left precuneus, left paracentral lobule, left supplementary motor area and right inferior frontal gyrus were highly reproducible. This limitation may be attributed to the relatively small sample size and the split-half analysis, which may have significantly reduced the statistical power of the group comparisons. Therefore, a leave-one-out validation was employed to test the reproducibility and robustness of the previous findings on fALFF and FC without losing statistical power. Specifically, we left one PNES patient out of the sample and performed the same group comparisons based on the permutated sample (i.e., 17 PNES vs. 20 healthy controls ), obtaining a total 18 two sample t-test images (*p* < 0.05, AlphaSim corrected). For each voxel in the *t*-test images, the number of tests where this voxel exhibited significant group differences was calculated as the reproducibility of the fALFF and FC differences between the two groups, respectively. The results showed that the difference in fALFF and FC patterns (Fig. 4B and Fig. 5B) between two groups were similar to that in the full group. Together, these results suggest a high reproducibility of our findings.

## Discussion

The present fMRI study investigated the resting state brain function by measuring the regional fALFF and inter-regional FC in patients with PNES. Patients with PNES showed significantly increased synchronous regional brain activity mainly in the dorsolateral prefrontal cortex (DLPFC), parietal cortices and motor areas, as well as decreased regional activity in the triangular IFG. Thus, our results add to literature suggesting that abnormalities of neural synchrony in PNES and extend these findings to the LFO domain. In addition, neural network deficits in PNES patients were revealed by seed analyses, including both increased and decreased FC. Moreover, the correlation analyses demonstrated a significant positive trending correlation between the FC values and the PNES frequency. These findings indicate that changes in the regional cerebral function are related to remote neural activity across a widely distributed brain functional network.

One cortical area where we observed significantly increased regional synchronous activity in PNES was the left PFC. Previous cerebral blood flow studies have demonstrated that PNES-related disorders exhibit altered cerebral activity in the prefrontal cortex^26^, which are believed to have implications for deficits in emotion regulation and executive function. Given that PNES is often accompanied by many diffuse psychological, psychiatric and somatoform symptoms^23^,^27^, the enhanced activation of PFC may be very likely to contribute to the impairment of executive control function and the manifestation of emotional triggers as somatosensory symptoms. Additionally, we noted that impairment of consciousness and reduced self-control are key features of most PNES (94% of cases in the reported sample). Both PFC and precuneus are components of the default mode network (DMN) associated with awareness^28^. A recent resting state networks study suggested that abnormalities in DMN may be important for the specific alterations of consciousness in PNES^29^.The increased spontaneous activity in PFC and precuneus in patients may therefore cause its awareness impairment or even loss. The precuneus, part of the DMN, is thought to be involved in self-consciousness and self-related mental representations during rest^30^. Increased regional fALFF in PNES was observed in the precuneus, and the functional integration of this region was also found to be abnormal. Our observations are also coherent with precuneus dysfunction that has been identified in patients with other conversion disorders^31^. These imaging findings provide converging evidence that suggest the function of PFC and precuneus in disrupting the normal integrative functions of consciousness in PNES.

We also observed hyperactivity in particular regions belonging to SMN, which is involved in self-initiated and involuntary muscle movements. It is important to note that PNES were characterized by stereotyped motor phenomenon. In our patients with PNES, several of the cases showed hypermotor or trembling movements of the extremities. The increased fALFF in SMN may thereby reflect altered motor control functions in PNES. These results are consistent with the findings from other work that showed abnormalities of the motor areas in PNES^17^. In a structural MRI study^32^, PNES patients exhibited abnormal cortical atrophy of the motor and premotor regions. In another study that employed the fMRI technique, van der Kruijs *et al*.^5^ found that increased FC was involved in the sensorimotor cortex. Therefore, our findings of increased spontaneous activity in the motor regions together with previous studies strengthen the view that the SMN plays an important role in the pathogenesis of PNES.

It is potentially interesting that PNES displayed significantly decreased fALFF in the triangular IFG. Pars triangularis is part of the ventrolateral prefrontal cortex (VLPFC) and has been shown to play a role in cognitive control of memory^33^. A previous study demonstrated that cognitive integrative functions for dealing with social stress and memory are impaired in patients with PNES^34^. A recent study employing a data-driven technique indicated lower long-range functional connectivity density (FCD) of the triangular IFG in PNES^35^. For the regional cerebral function perspective, our findings provided new evidence for the impairments of the working memory in patients with PNES. More task-related studies are needed to identify the neuromechanism that contribute to altered spontaneous activity as observed in the current study.

Alterations of FC in PNES have attracted attention as a potential network level substrate of this disorder. Increased connectivity within the frontal-parietal and SMN has been reported^17^,^29^. A study involving the use of EEG synchronization found that PNES appeared to be associated with decreased prefrontal and parietal synchronization^4^.These inconsistent findings may be due to the different methodologies and parameters used, as well as the different characteristics of the participants. In this study, we demonstrated that the widespread altered inter-regional connectivity beyond the regional activity alterations in the PFC and motor areas was also related to PNES.

The altered connectivity pattern of PFC may provide new insights into how prefrontal areas affect motor system in patients with PNES. Although the PNES showed decreased coactivation in VLPFC, the DLPFC displayed enhanced connectivity with motor regions. A task-related study has found that conversion paralysis patients exhibited altered connection between DLPFC and various sensorimotor nodes during the formation of action plans, whereas ventromedial PFC was not functionally connected to the motor system^36^, thereby indicating a distinct connectivity patterns for different parts of the PFC. DLPFC plays an important function in the circuitry of emotional control, and it monitors attention resources and guides emotional responses^37^. The involvement of increased DLPFC-SMN connection is consistent with previous fMRI studies that have identified dysfunctional connectivity^5^ and increased long range FCD^35^ between emotional regions and SMN in PNES. As a result of this alteration, there is a failure for the PNES in the coordination and balance of different mental and somatic functions. Another main observation is the increased connectivity between ACC and SMA, and the enhanced connectivity is positively correlated with the frequency of PNES. The conversion disorder has been hypothesized to affect the abnormal inhibition of motor systems by limbic regions^38^.The proposed interaction model between limbic and motor control regions is consistent with previously increased amygdala connectivity with SMA in patients with conversion disorder^26^. Our results may add to these findings and suggest an altered functional organization that involves the limbic regions and the sensorimotor cortex in PNES. ACC is an important region functionally connected with SMA^39^, and the connectivity between them may be involved in conveying information about negative emotion to the motor areas associated with executing goal-directed behavior^40^. Accordingly, it is reasonable to speculate that the ACC is a meeting place between cognitive and motivational processes; hence, altered connectivity between ACC and SMA in PNES may generate uncontrolled motor output. Furthermore, the altered connectivity between the SMA and ACC is correlated with the frequency of PNES, thereby suggesting a skewed integration of synchronous activity predisposed to the occurrence of PNES episodes. The results may be important therapeutically, such as selectively reducing or modulating FC, which may improve the disrupt balance and so prevent PNES events.

This study also has certain limitations. First, the sample size was modest and a relatively weak correction (AlphaSim program) was used for multiple comparisons in our study. A larger sample is needed to replicate and support these results. Second, this study employed a cross-sectional design and is thus can not provide a test-retest reliability^41^,^42^,^43^. A follow-up design could provide longitudinal data to clarify abnormal spontaneous neural activity and functional network deficits in PNES. In addition, although ALFF is believed to reflect spontaneous neural activity, there is a lack of understanding about its exact physiological nature. Finally, no significant correlation was observed when we examined the relationship between fALFF and FC in the PNES group. We speculate that the sample size was not enough to provide sufficient evidence to describe the relationship between them. A further study is required to better elucidate that whether the changes in the regional neuronal activity could cause an alteration in the inter-regional functions of patients with PNES.

Taken together, the findings of abnormal fALFF and FC in PNES were actually derived from the examination at two different analytical levels. The higher fALFF in the PFC and sensorimotor system suggests the regional abnormalities of neural synchrony in PNES, whereas the altered FC in the fronto-parietal cortex and motor regions indicates that the alterations in the regional cerebral function is related to remote inter-regional functional network deficits. Accordingly, we claim that the dysfunction of the fronto-parietal cortex and SMN may be important factor that could affect the emotion regulation, executive control and motor function of PNES. Overall, our findings from both regional neural function and inter-regional network perspective provided novel evidence to investigate the pathophysiological mechanisms of PNES.

## Methods

### Participants

The subjects were recruited from our previous studies^17^. A total of 20 patients with PNES (7 males, mean age: 19.65 ± 7.56 years) and 20 controls (8 males, mean age: 21.85 ± 1.70 years) were recruited from West China Hospital. No significant difference in age (*t* = 1.27, *p* = 0.12) or sex (*x* *=* 0.11*, p* *=* 0.74) was found between the groups. PNES was diagnosed by the experienced neurologists using clinical descriptions of symptoms and long-term video-EEG monitoring based on the recent recommendations^44^,^45^. The detailed inclusion and exclusion criteria are described in the Supplementary materials. Four of the 20 patients were taking antiepileptic drugs before the diagnosis of PNES. All drugs were discontinued at least two weeks prior to MRI examination. The demographic and clinical characteristics for all patients are presented in Table 1. PNES frequency was assessed according to the reports of the patient or their family members. The median frequency was 2.5 per month (with a range of one per week to eight per week). Two patients have a long duration of disease with 18 years, because of which the two patients were excluded from the analysis to ensure the illness conditions of the patients are similar as possible. The HC neither had neurologic/psychiatric disorders evaluated using the SCID-Non-Patient Version, nor did they take any psychotropic medication within the past six months. This study was approved by the local Ethics Committee of West China Hospital and was carried out in accordance with the approved guidelines. All participants gave informed consent to participate in the investigation.

### Data acquisition

Magnetic resonance images were acquired using a 3T Siemens Trio scanner (Erlangen, Germany) at the West China Hospital of Sichuan University in Chengdu. Participants were instructed to remain motionless and relax with their eyes closed without falling asleep. Meanwhile, foam pads and earplugs were used to reduce head motion and scanner noise. The resting state fMRI images were obtained by using an echo planar imaging sequence for a total of 205 volumes with the following parameters: TR = 2000 ms; TE = 30 ms; flip angle = 90°; field of view = 240 × 240 mm^2^; acquisition matrix = 64 × 64; voxel size = 3.75 × 3.75 × 5 mm^3^, no slice gap; 30 axial slices. A total of 205 volumes were acquired for each participant.

### Data preprocessing

Data preprocessing was performed using the SPM8 package (http://www.fil.ion.ucl.ac.uk/spm) and included the removal of the first five volumes, corrected for time delay between slices and head motion, spatial normalization to the Montreal Neurological Institute (MNI) space and smoothed by convolution with an 8-mm full-width half-maximum Gaussian kernel. Finally, the six head motion parameters as nuisance covariates were regressed to reduce the effects of head motion, because a recent study demonstrated that head motion had a confounding effect on ALFF analysis^46^.

### fALFF Calculation

The fALFF analysis was carried out using the REST software (http://resting-fmri.sourceforge.net). After preprocessing in SPM, a linear trend was removed. To calculate ALFF measure at each voxel, the time series were transformed to the frequency domain by using fast Fourier transform. The power spectrum was then computed and square root-transformed at each voxel. Considering that the power of a given frequency is proportional to the square of the amplitude of this frequency component, the square root was computed at each frequency of the power spectrum and the averaged square root was obtained across 0.01–0.08 Hz at each voxel. This averaged square root was taken as the ALFF^13^. fALFF is the fraction of ALFF in a given frequency band to the ALFF over the entire frequency range detectable in the given signal^14^. fALFF of each voxel was computed for each subject, and it was further divided by the global mean value to reduce the global effects of variability across participants^13^.

### Functional connectivity analysis

FC was examined using a seed voxel linear correlation approach^47^,^48^. As discussed later, significant changes in fALFF measurements of PNES were detected in 4 clusters after AlphaSim correction (Table 2). We employed a seed-based approach whereby the mean time series for each participant were extracted from these clusters by masking the Automated Anatomical Labeling (AAL) template^49^. This way, the resulting six masks were defined as seeds for further FC analyses. To perform the analyses, the time series of fMRI data for each voxel were filtered by applying a bandpass filter (0.01–0.08 Hz). Each time series was further corrected for the effect of six head motion parameters by linear regression to reduce the influence of head motion. Recent studies have showed that functional connectivity analysis is sensitive to gross head motion effects^50^,^51^; hence, the mean frame-wise displacement (FD) was calculated to further determine the comparability of head movement across groups^50^. The largest FD of all subjects was less than 0.2 mm. Two-sample *t*-test showed that there was no significant difference in FD between the two groups (0.116 ± 0.210 for HC and 0.117 ± 0.041 for PNES; *p* = 0.930). In addition, the averaged signals from the specific CSF and white matter masks were regressed based on the previous resting state fMRI studies^52^,^53^. Since removing the global signal may introduce a shift in the distribution of correlation coefficients (mainly the presence of negative correlations) and make biological interpretation ambiguous^54^,^55^, we did not include the global signal as a covariate variable. Then, a reference time series was extracted by averaging the filtered signal of all voxels within the six regions showing a significant change in fALFF values. For each seed, a linear correlation analysis was conducted by calculating the correlation coefficient between the time series of the seed region and the time series of each voxel across the whole brain. The resultant r values were converted to z maps using Fisher’s *r*-to-*z* transformation^56^ to improve the Gaussianity of their distribution. Finally, we obtained 6 *z*-score maps of the seed to all other brain voxels for each subject.

### Statistical analysis

The statistical analyses involved in a comparison between PNES patients and healthy controls in terms of (1) regional cerebral fALFF values, (2) inter-regional FC between seeds and the rest voxels of the brain. We first performed a random effect one-sample *t*-test on all individual fALFF maps for the two groups respectively. This procedure produced significant fALFF statistical maps for both groups. The significance level for each group was set at *p* < 0.01 with FDR correction. We then made a mask that combines the two fALFF statistical maps to analyze the differences in fALFF between patients and controls. According to a previous study^14^, such a mask can remove the influence of large vessels and constrain our analyses within regions generating potentially meaningful LFOs. These same analyses were performed on FC maps, and the threshold of one-sample *t*-test was set at *p* < 0.01 corrected with FDR. Two-sample *t*-tests with age and gender as covariates were performed to identify the regions that showed significant differences in fALFF and FC between the two groups. The resulting statistical fALFF maps were corrected for multiple comparisons to a significant level of *p* < 0.05 by combining the height threshold of p < 0.01 with a minimum cluster size of 75 voxels, using the AlphaSim program in the REST software (http:/resting-fmri.sourceforge.net).The same statistical approaches as those described for the analyses of fALFF were used for thresholding the FC maps. All the statistical maps were corrected by AlphaSim at a significant level of *p* < 0.05 by combing height threshold of *p* < 0.01 with a minimum cluster sizes of 116, 115, 162, 150, 79 and 157 voxels, respectively. Coordinates were reported in the Montreal Neurological Institute space provided in SPM8.

Three Pearson correlation analyses were performed: 1) fALFF values in regions with alterations were correlated with PNES frequency (the average number of episodes that patients experienced for a month); 2) FC in regions with significant group differences was correlated with PNES frequency; 3) fALFF values in those regions with abnormalities were correlated with FC. Pearson correlation was calculated with age, gender and duration of disease as covariates to be regressed. The threshold of *p* < 0.05 was considered to be significant for these correlation analyses.

## Additional Information

**How to cite this article**: Li, R. *et al*. Altered regional activity and inter-regional functional connectivity in psychogenic non-epileptic seizures. *Sci. Rep*. **5**, 11635; doi: 10.1038/srep11635 (2015).

## Supplementary Material

Supplementary Information

## Figures and Tables

**Table 1:** Demographic and clinical characteristics of PNES.

Patient	Age	Gender	Duration	Frequency (times/m)	Type of symptoms	Previous Treatment*
1	34y	F	18y	2	Unresponsiveness/eye closure	None
2	17y	F	2y	8	Unresponsiveness/eye closure/hyperventilation/bod rigidity	None
3	38y	F	18y	3	Unresponsiveness/hypermotor EX	None
4	23y	F	8y	1	Unresponsiveness/eye closure/hyperventilation/bod rigidity	None
5	13y	F	1m	5	Unresponsiveness/eye closure	None
6	20y	M	2y	2	Unresponsiveness	VPA
7	14y	F	2m	3	Unresponsiveness	None
8	17y	F	2m	4	Unresponsiveness/eye closure/bod rigidity/trembling EX	None
9	14y	M	5m	5	Unresponsiveness/vocalization	VPA
10	16y	F	2y	0.3	Unresponsiveness/hyperventilation/hypermotor EX	None
11	17y	F	4m	4	Unresponsiveness/hyperventilation	None
12	21y	F	8m	4	Unresponsiveness/hypermotor EX	None
13	21y	M	1m	1	Eye closure/hyperventilation/bod rigidity	None
14	13y	M	1y	1	Unresponsiveness/eye closure/hyperventilation	None
15	13y	F	1y	0.5	Unresponsiveness/eye closure/hyperventilation	None
16	35y	M	15d	4	Unresponsiveness/eye closure/hypermotor EX	None
17	16y	F	3y	4	Unresponsiveness	CBZ
18	20y	M	2y	0.5	Unresponsiveness/hypermotor EX	VPA
19	13y	M	1m	1	Unresponsiveness	None
20	18y	F	7m	2	Unresponsiveness/eye closure	None

Notes: F, female; M, male; d, day; m, month; y, year; Hypermotor EX, Hypermotor movements of the extremities; Trembling EX, Trembling of the extremities; VPA, valproate; CBZ, carbamazepine.

^*^All the drugs were discontinued at least two weeks before MRI examination.

**Table 2:** Regions that showed significant differences in fALFF between controls and patients with PNES.

					MNI coordinates		
	Brain regions	BA	Cluster size	Peak t-value	X	Y	Z
**PNES>HC**							
Cluster 1	L Superior frontal gyrus	9	118	5.41	−12	27	48
Cluster 2	L Precuneus	7	107	4.99	−12	−42	3
Cluster 3	L/R Sensorimotor area	3/4/6	587	4.78	−15	−36	54
	L Paracentral lobule						
	R Postcentral gyrus						
	L Supplementary motor area						
**HC>PNES**							
Cluster 1	R Triangular inferior frontal gyrus	46	90	−4.21	57	30	21

Notes: Results were corrected by AlphaSim(all the clusters survived *p *< 0.05, a combined threshold of *p *< 0.01 with a minimum cluster size of 75 voxels).The coordinates were showed in the Montreal Neurological Institute (MNI) standard space. fALFF, fractional amplitude of low-frequency fluctuations; PNES, psychogenic non-epileptic seizures; HC, health controls; BA, Brodmann area; L, left; R, right.

**Table 3:** Regions that showed significant differences in FC between controls and patients with PNES.

					**MNI coordinates**		
	**Brain regions**	**BA**	**Cluster size**	**Peak t-value**	**X**	**Y**	**Z**
**L Superior frontal gyrus**							
Cluster 1	R Paracentral lobule	2	226	3.68	36	−33	63
Cluster 2	L Inferior frontal gyrus, orbital	47	179	4.06	−21	33	−12
**L Precuneus**							
Cluster 1	L Superior frontal gyrus, medial orbital	10	194	3.19	0	51	−9
**L Paracentral lobule**							
Cluster 1	L Middle frontal gyrus	9	184	4.73	-33	36	42
Cluster 2	R Supplementary motor area	7/31	641	3.96	6	−21	48
	L Precuneus	7		3.88	−6	−51	48
	R Precuneus	7		3.64	3	−39	54
	L Median cingulate gyrus	31		3.55	−9	−30	45
	R Median cingulate gyrus	31		3.86	9	−24	42
**R Postcentral gyrus**							
Cluster 1	L Middle frontal gyrus	9	257	5.28	−33	33	42
Cluster 2	L Superior parietal lobule	7	176	3.57	−33	−72	54
**L Supplementary motor area**							
Cluster 1	L Middle frontal gyrus	9	111	4.61	−33	33	42
Cluster 2	R Anterior cingulate gyrus	32	336	3.21	9	42	−9
	L Anterior cingulate gyrus	32		2.66	−6	36	9
**R Triangular inferior frontal gyrus**							
Cluster 1	R Middle frontal gyrus	9	202	−4.56	39	15	60

Notes: Results were corrected by AlphaSim(all the clusters survived *p *< 0.05, a combined threshold of *p* < 0.01 with a minimum cluster sizes of 116,115,162,150,79 and 157 voxels, respectively ).The coordinates were showed in the Montreal Neurological Institute (MNI) standard space. PNES, psychogenic non-epileptic seizures; Hem, hemisphere; BA, Brodmann area; L, left; R, right.
