# Supplementary material for: Altered regional activity and inter-regional functional connectivity in psychogenic non-epileptic seizures
Source: Sci Rep. 2015 Jun 25;5:11635. doi: 10.1038/srep11635 (PMC4480007; doi:10.1038/srep11635)
Supplement: Supplementary Information [file srep11635-s1.pdf]

Altered regional activity and inter-regional functional connectivity in psychogenic non-epileptic seizures

Supplementary materials

Participants

PNES met the following inclusion criteria: 1) at least one single typical episode was recorded by video EEG, and EEG did not show any epileptiform discharge or ictal slowing; 2) patients have no history of neurological disease; 3) patients have no obvious abnormality in routine structural MRI examinations. The exclusion criteria were: 1) patients with neurological comorbidity (e.g. epilepsy); 2) patients with malingering, or any psychiatric disorders (e.g. mood and anxiety disorders, schizophrenia and psychosis). Here, the diagnosis of malingering or psychiatric disorders was determined by two attending psychiatrists using the Structured Clinical Interview for DSM-IV (SCID)-Patients Version and their scores on the Positive and Negative Syndrome Scale, Hamilton Anxiety Rating Scale and Hamilton Depression Rating Scale. Only patients with a diagnosis of definite PNES were included in this study.
